# The Effectiveness of Structured Neurologic Music Therapy on Phonation and Breathing Function Following COVID-19

**DOI:** 10.7759/cureus.87374

**Published:** 2025-07-06

**Authors:** Christina V Oleson, Samuel W Onusko, Michelle S Djohan, Mary J Roach, Carol S Mizes, Dwyer B Conklyn

**Affiliations:** 1 Physical Medicine and Rehabilitation/Spinal Cord Injury Medicine, MetroHealth Rehabilitation Institute/Case Western Reserve University, Cleveland, USA; 2 Physical Medicine and Rehabilitation, MetroHealth Medical Center/Case Western Reserve University, Cleveland, USA

**Keywords:** covid-19, diaphragmatic breathing, music therapy, post-acute sequelae of covid, respiration, therapeutic singing, vocal intonation therapy

## Abstract

Objective

To analyze the effectiveness of neurologic music therapy (NMT), consisting of diaphragmatic breathing (DB), vocal intonation therapy (VIT), and therapeutic singing (TS), on sustained phonation and oxygen use during recovery from the recent coronavirus disease 2019 (COVID)-19 or post-acute sequelae of severe acute respiratory syndrome coronavirus 2 (SARS‑CoV‑2) (PASC).

Methods

The study was designed as a retrospective cohort study. Sixty-nine adults admitted to acute inpatient rehabilitation with recent COVID-19 or PASC received standard physical, occupational, speech, and respiratory therapies. All were given a graded exhalation device with vibratory stimulation to help strengthen the muscles of expiration and clear secretions. Bronchodilators were administered as needed. Supplemental oxygen was prescribed to maintain saturations >92%. Thirty-three individuals also chose to receive NMT 1-3 times weekly for the duration of their inpatient rehabilitation hospitalization. The main outcome measures were changes in oxygen demand during rehabilitation and breath control, defined as sustained phonation (expiration) from a single breath. Secondary outcomes were discharge disposition and NMT participant experience.

Results

Compared with the non-NMT group, NMT participants required significantly more oxygen during activity at both admission (p=.010) and discharge (p=.007). Within the NMT group, significant improvement in oxygen demand was observed from admission to discharge, both at rest (.7833L reduction, p=.003) and during activity (.8500L reduction, p=.003). The degree of improvement in the need for oxygen also showed differences between the two groups. Compared with non-NMT participants, those receiving NMT demonstrated a significantly greater reduction in activity-based oxygen use during rehabilitation (p=.014, Cohen’s d 2.06). Despite a longer duration in acute care than the non-NMT group, the NMT participants had an equivalent number of days in inpatient rehabilitation and a higher rate of home discharge (p=.018). Positive correlations were found in changes in phonation abilities, measured by the number of NMT sessions completed (p=.021) and by minutes of NMT received (p=.009). The majority of participants rated their experience with this program as positive.

Discussion

Proper DB with VIT and TS can have a meaningful effect on sustained phonation, breathing tolerance, and vocalization, resulting in participants reporting fewer pauses for air while speaking. NMT was considered a positive experience that also potentially contributed to the reduction in oxygen dependence after COVID-19.

## Introduction

Nearly all components of the respiratory system, from the conducting airway passages to the vascular endothelium, pulmonary blood flow, and neuromuscular function, can be impacted by acute infection with severe acute respiratory syndrome coronavirus 2 (SARS‑CoV‑2), the virus responsible for coronavirus disease 2019 (COVID-19) [1]. Therapeutic singing has been shown to improve respiratory muscle function, breathing efficiency, circulatory changes, and social and emotional well-being [2]. Kang and colleagues described how persistent singing utilizes the cardio-respiratory system, thereby strengthening respiratory muscles and facilitating the coordination of breathing [2]. This investigation will evaluate how certain aspects of neurologic music therapy can be incorporated into a comprehensive rehabilitation program, focused on improving respiratory function in persons with recent coronavirus disease 2019 (COVID)-19 or with post-acute sequelae of SARS-CoV-2 (PASC).

Neurologic music therapy (NMT) adopts patterned exercise techniques with specific elements of musical construction (rhythm, tempo, and pitch) in combination with the non-musical properties of vocal output, speech, and physical movements to improve vocalization [3]. The neurologic aspect refers to the reinforcement of patterns learned that enhance motor, cognitive, and speech pathways in the brain and central nervous system such that repeated patterns may be performed with greater ease and less energy expenditure. According to Thaut, rhythm and music are able to “prime” motor, cognitive, and speech pathways in the brain at a subconscious level, including those responsible for breathing patterns, and build new neuropathways that expedite these physical and cognitive processes [3]. The techniques involved in NMT stand in contrast to general music therapy, which seeks to address the emotional, physical, and mental well-being of an individual learner.

Our project will focus on three aspects of therapeutic intervention that directly or indirectly involve musical technique: diaphragmatic breathing (DB), vocal intonation therapy (VIT), and therapeutic singing (TS). DB should begin with a deep breath through the nose that can be felt in the abdomen, without the use of accessory chest or shoulder muscles. Engaging the diaphragm in deep breathing involves inhaling through the nose slowly with sequential involvement of deeper respiratory passages in the lung, with the eventual activation of abdominal muscles located below the diaphragm muscles [4]. The brain, cardiovascular, respiratory, and gastrointestinal systems can be affected by DB through regulation of the autonomic nervous system with additional effects on the central nervous system [5]. Diaphragmatic breathing and pacing between breaths are key components of singing.

VIT is an NMT technique that uses vocal exercises to train, maintain, develop, and rehabilitate aspects of voice control. Structural, neurologic, physiologic, and functional abnormalities of the voice apparatus may benefit from VIT, including vocal dynamics, inflection, pitch, breath control, and timbre [6].

TS addresses a wide spectrum of functions in a more general and undifferentiated manner than the other NMT methods by combining outcomes learned through the speech and language NMT techniques into an individualized experience, incorporating one’s preferred music. Breath control, articulation, DB, or vocal intensity can be applied while singing a complete song [7].

Aspects of music therapy have been evaluated in viral illnesses similar to SARS-CoV-2. Wang et al. extrapolated information from previous studies of persons with COVID-19, persons without COVID-19 but receiving pulmonary rehabilitation, and persons with previous severe Middle Eastern respiratory syndrome of coronavirus (MERS-CoV) [8]. The authors recommended deep DB, engaging diaphragmatic and abdominal musculature while limiting the use of accessory muscles of respiration. Because vocal music uses timed breathing techniques, authors believed singing could be a component of therapy for those in the mild stage of various coronavirus infections. We suspect these techniques may also be helpful in rehabilitation after acute SARS-CoV-2.

According to the American Academy of Physical Medicine and Rehabilitation’s Long COVID Guidelines, long-term effects of coronavirus infection can include fatigue, shortness of breath, cough, joint pain, and chest pain [9]. Since little has been published on the effect of NMT in patients with COVID-19, support for instituting this type of intervention can be drawn from similar techniques in other disorders that can adversely affect pulmonary function. Given the profound muscle weakness observed in patients recovering from prolonged hospitalization and the critical illness seen after an SARS-CoV-2 infection, developing a therapy protocol to address the pulmonary deficits in PASC was also central to our study purpose.

The effect of vocal exercises and singing on impaired breathing function in people with neurological deficits and pulmonary dysfunction has been extensively explored in the literature. Critical illness myopathy and polyneuropathy result in notable weakness of muscles of expiration following COVID-19, particularly after a prior, prolonged intensive care unit (ICU) admission [10,11]. Neurovascular conditions of the brain or spinal cord, including stroke, spinal cord infarction, inflammatory conditions of acute inflammatory demyelinating polyneuropathy (AIDP), or transverse myelitis, have been reported after COVID-19 [10-14]. An NMT program designed to improve expiratory muscle function may benefit this population based on prior investigations.

Positive effects using vocal exercises and singing were reported in a randomized controlled trial (RCT) of 24 persons with tetraplegia [15]. Conducted in a hospital setting, this study demonstrated that tetraplegic individuals in the singing intervention group exhibited increased activation of respiratory muscles (including accessory muscles), compared with tetraplegic persons receiving standard therapy alone. Subjects also demonstrated a dose-dependent improvement in expiratory function that correlated with vocal intensity and endurance.

A randomized controlled trial (RCT) with incomplete spinal cord injury individuals evaluated the effectiveness of VIT on respiratory function and vocal quality [16]. The authors reported relative improvement in the intervention group at six weeks but statistically significant differences after 12 weeks. The intervention group also demonstrated a significant increase in quality-of-life measures.

Our study focused on the respiratory benefits of DB and vocal exercises in the treatment of people with a recent history of COVID-19, with or without defined PASC. Our objectives were to analyze the effect of NMT on oxygen use, phonation, discharge disposition, and participant experience with the protocol. Based on our population of persons with both recent SARS-CoV-2 infection and sequelae involving neurologic, neuromuscular, or musculoskeletal deficits, we believe an NMT program may be helpful in the recovery of both intraparenchymal and extraparenchymal breathing difficulties arising from COVID-19. We further anticipated the majority of participants would rate the NMT protocol and overall experience positively. At the time of this writing, there were no published articles measuring the effects of music therapy techniques to improve breathing function in those with recent COVID-19 or PASC. The goal of our study was to gather enough preliminary data through a chart review to develop a more robust investigation of the use of NMT to enhance breathing tolerance and vocalization following COVID-19.

The preliminary results of this study were previously presented in poster format at the Association of Academic Physiatrists Annual Meeting in February 2024.

## Materials and methods

Study design and participant selection

Initial approval for the creation of a database of persons with rehabilitation needs after acute COVID-19 was obtained on November 23, 2020, from the MetroHealth System’s Institutional Review Board (approval number 20-00570. Our study drew subjects from this database and was given specific approval for the inclusion of music therapy clinical notes in the existing database on November 12, 2021.

A retrospective cohort design was chosen for two reasons. First, we anticipated the inability to control unforeseen challenges and barriers with a prospective design during the height of the pandemic. Second, it was considered unethical to offer treatment that might potentially benefit persons recovering from COVID-19 to some individuals but not others. After having received approval for this study, we began screening charts of persons who presented for admission to acute rehabilitation from April 1, 2020, through December 31, 2021 with continued follow up of inpatient hospitalization through March 31, 2022. To be included, participants needed a diagnosis of acute COVID-19 within the preceding six months and must have been admitted to rehabilitation from acute care or long-term acute care, not from a home setting, assisted living, or skilled nursing. Individuals needed to be experiencing some residual symptoms of COVID-19 covered by the series of consensus guidance statements on PASC by the American Academy of Physical Medicine and Rehabilitation to be eligible for the study [9]. The full inclusion and exclusion criteria are given in Table 1. 

**Table 1:** Inclusion and exclusion criteria NMT: neurologic music therapy; LTAC: long-term acute care; SNF: skilled nursing facility

Inclusion Criteria
Minimum age 19
Acute COVID-19 infection in the preceding 6 months with residual symptoms in at least one organ system or one systemic complaint as defined in reference [9]
Current admission to acute inpatient rehabilitation with immediately preceding stay in acute care, LTAC, or acute care to SNF combination
If a tracheostomy is present, tolerance to a one-way speaking valve or cap for the duration of the NMT training session
Oxygen permitted if the requirement was at or below 40% at rest via a tracheostomy collar with a speaking valve or nasal cannula
Able to converse with music therapist to respond verbally to therapy satisfaction questions
Attendance at a minimum of 3 NMT sessions spread over 2 weeks
Minimum length of time in inpatient rehabilitation: 10 days
Able to sit upright for one hour at a time for NMT sessions with symptoms of dizziness or hypotension
At least 14 days post-COVID-19 infection
Exclusion Criteria
Ventilator dependence
Need for open tracheostomy during an NMT session
History of pulmonary lobectomy, partial or complete
Requirement for greater than 40% oxygen at rest (via a nasal cannula or tracheostomy collar/speaking valve combination)
Interrupted inpatient rehabilitation stay of more than 7 days (the first or second part of inpatient rehabilitation could be considered, but the halves would not be combined)
Inability to understand the NMT protocol due to a language barrier or severely impaired cognition
Prior experience as a professional singer or wind instrument player
Vocal cord dysfunction

The following information was obtained from a chart review: age, gender, race, ethnicity, length of stay in acute care and or long-term acute care, length of stay in rehabilitation, and discharge disposition. Oxygen use at rest and during activity while in acute inpatient rehabilitation was gleaned from the medical record at three time points: admission to inpatient rehabilitation, midpoint of the rehabilitation stay, and at discharge from rehabilitation. The amount of oxygen during therapy was recorded separately from the amount of oxygen needed at rest at all three time assessments. Oxygen requirement documentation at any measurement point was cross-checked between respiratory therapy data recordings and the progress notes of physicians, nurses, and therapists. In addition to comparing oxygen requirements between the NMT and non-NMT groups at the beginning and conclusion of rehabilitation, we also wanted to compare progress between groups during rehabilitation. Intubation and ventilator use prior to rehabilitation, as well as the continued presence of a tracheostomy tube during rehabilitation, were recorded. Any heart rate limitations that needed accommodation with appropriate rest breaks during therapy sessions were recorded. During these sessions, patients were monitored with a pulse oximeter machine to ensure saturations were maintained and heart rate remained within safe limits. All individuals in our study groups were offered a program of NMT, but participation was voluntary. Our sample was divided between persons who received NMT and those who declined participation.

Present, current, or preexisting neurological conditions were documented, including a high clinical suspicion of critical illness polyneuropathy or myopathy. Admitting diagnoses of NMT and non-NMT participants were obtained to the extent documented in the medical record. The comorbidities collected included chronic obstructive pulmonary disease (COPD), diabetes mellitus, current smoking tobacco use, obesity defined as body mass index >30, and asthma. Both the preexisting medical issues and comorbidities, even if well-controlled, were obtained in an effort to establish equivalency between groups at baseline. The source of this data was the past medical history portions of the record and the admitting history and physical exam. The inpatient admission evaluation is obtained and verified by the rehabilitation physicians and includes confirmation of stated prior medical conditions. We selected the above comorbidities from a broader list of conditions commonly found among post-COVID patients because the selected conditions could influence the need for supplemental oxygen, rehabilitation length of stay, and ultimate discharge disposition.

The study size was determined by the number admitted during the study period. We chose to end the inclusion of those newly admitted after December 31, 2021, all of whom were discharged by March 31, 2022. Subsequent to the latter date, new music therapy staff who joined our program suggested changes to the NMT protocol. Such changes had the promise to benefit patients but would introduce bias for individuals included after that date. Groups were divided based on their choice of participating in NMT or electing to only pursue the standard combination of one-hour physical, occupational, and speech therapies, to meet the three-hour daily requirement of acute inpatient rehabilitation facilities in the United States. Bias was controlled to the extent possible in a retrospective cohort by excluding patients with significant physical limitations and those whose backgrounds in music training would produce falsely elevated performance results. People fitting the above descriptions still received NMT as patients admitted to our institution, but they were excluded from this retrospective cohort. Because all patients may be depressed or anxious after acute infection with COVID-19, rehabilitation therapy was offered to all participants of both groups. The frequency of counseling sessions varied from one to two times per week based on clinical need as designated by the rehabilitation psychologist, physician, and therapists.

Respiratory therapy for all participants

As part of standard clinical care following COVID-19 and other similar respiratory illnesses, each patient initially receives scheduled nebulizer treatments. Supplemental oxygen to meet daily needs and the physical demands of full rehabilitation therapy participation is given to all individuals based on their unique clinical condition, similar to those described in the collaborative consensus guidance statement from Maley et al. [17]. Those who did not need bronchodilators regularly received such treatment only upon request. Some patients required supplemental oxygen at all times, while others needed oxygen only during activity or sleep. To optimize tolerance and participation in rehabilitation, individuals received the necessary amount of supplementary oxygen, which resulted in maximal participation in activities and facilitated full rehabilitation participation. As described by Wang et al., supplemental oxygen allows partial offloading of respiratory muscles during more strenuous physical activities than previously undertaken by an individual at that point in rehabilitation [8]. To assist with expiratory strengthening and with airway clearance of retained secretions in deep passages of the bronchial system, a graded exhalation device with vibratory stimulation was given to all patients for use (VibraPEP, Tri-anim Health Services, Dublin, OH). This is an acapella-type device with a variable degree of selected resistance, often used to assist with mucus clearances in those with bronchiectasis, COPD, cystic fibrosis, and various forms of atelectasis [18]. Patients were asked to use this device for a minimum of 15 repetitions three times daily, but longer trials and higher frequency of use were encouraged. Except in cases in which an individual lacked hand function and required direct assistance with device use, nursing and respiratory staff were unable to confirm and document precise repetitions and frequency of use of the vibratory stimulation device, but did leave general documentation of “left within reach at the bedside” or “used during shift.”

The NMT protocol

For interested participants, a structured NMT protocol involving diaphragmatic breathing, vocal intonation therapy, and, for higher-functioning participants, therapeutic singing, in addition to their standard three hours of therapies (physical, occupational, and speech). Music therapy notes were accessed from the medical record. Patients were seen one to three times per week for 30-60 minutes per session, depending on their therapy schedules and availability. If seen only once a week, the individual received 60 minutes of NMT. Only those with at least three total hours of NMT over at least three separate dates were included in our study group. Pre- and post-test measurements of sustained phonation in seconds were recorded during training sessions by the music therapy intern. Sustained phonation may be considered the ability to extend the expiratory process and utilize breath efficiently while vocalizing a note or sound [2]. Patients were asked to maintain or “sing” a phonation, usually on the syllable “meee” or “mooo” for as long as they could in one breath. This was measured with a stopwatch. If the patient had difficulty with their breath coordination or control, they were given up to four additional attempts. The minimum number of attempts per patient was two. The test was performed prior to the first treatment session, repeated weekly to monitor progress, and then again at the end of their treatment time in rehabilitation. The initial and discharge times in phonation were compared, and the difference was calculated. The phonation outcome was measured in seconds. Interim phonation data were recorded only if the music therapist anticipated being away for the following week and there were concerns that a participant might be discharged early. In such cases, the data taken on the interim evaluation were used. Aside from special situations, interim “testing” data of phonation were not obtained because week-to-week gains could be slow, and we feared patients would feel they were “being tested” rather than receiving therapy. We recognized that some participants may not achieve functional gains from the final stage of the NMT protocol, specifically TS. Reasons for not reaching this milestone were vocal weakness, poor endurance, or lack of time prior to discharge. In these cases, the therapist still did some TS for engagement even if the participant did more listening or humming to songs than true vocalization. The authors felt that even time-limited modified TS might improve the motivation and mood of participants.

Music therapy training procedure

The therapist or music therapy intern demonstrated each step prior to the participant attempting to do each step. Sometimes, several demonstrations were needed. 

Diaphragmatic Breathing (DB)

The therapist demonstrated proper DB while standing sideways in front of individuals. Participants observed proper abdominal breathing, noting the duration of the inhale phase. The music therapist explained not to lift shoulders or chest, but rather to inhale through the nose and exhale through the mouth. A few slow deep breaths were demonstrated.

Vocal Intonation Therapy (VIT) Exercise #1

Participants were then asked to breathe the same way, along with the therapist, as the therapist counted three seconds for each inhale and three seconds for each exhale. If individuals demonstrated a stronger pre-test, then the counting started at four or five seconds. As participants became stronger in their therapy sessions, this counting increased. As they became stronger, individuals were encouraged to undertake a longer exhale than inhale, making sure to complete the expiratory process as much as possible.

Vocal Intonation Therapy (VIT) Exercise #2

The “ho, ho, ho” exercise: Participants were instructed to take a deep breath and then say ‘ho, ho, ho” with a quick break in between each utterance [6]. In music, this is called “staccato.” The pattern should be accomplished in one breath if possible and from the abdomen, not the throat. This helps to engage or contract the diaphragm. The process of abdominal contraction should be visible. Patients were instructed to do this a few times and as loudly as they could to improve muscular strength and vocal intensity. 

Vocal Intonation Therapy Exercise (VIT) #3

The above speaking exercise was translated into a musical exercise. Participants were instructed to sing “ho, ho, ho” in a triad, up and down in pitch, with staccato application. A musical example would be singing the word “ho” on each note C, E, G, E, C. The therapist generally started at the patients’ mid-to-lower range. Then, the therapist would increase the pitch by half-steps and repeat the sequence until patients were almost at the top of their range but still singing comfortably. This process was repeated, moving down in pitch by half-steps, to the lower end of the patients’ range. Depending on the condition of each patient, there were times when it was necessary to take breaks or do the process more slowly and to assist patients in taking deeper breaths at a brisk pace but avoiding hyperventilation. 

Vocal Intonation Therapy Exercise (VIT) #4

Participants were instructed to sing “me -yah - me” on one note, starting soft on “me” and gradually becoming louder on “yah” then gradually becoming softer on “me.” This step should take three to five seconds. Again, the therapist started at each individual’s mid-to-lower range, increasing the pitch by half-steps until participants were at the higher end of their range. The process was repeated by lowering the starting pitch by half-steps and continuing until they were near the lower end of their range. 

If the therapist felt patients needed to repeat any of these exercises due to poor pulmonary condition, lack of time in music therapy, or the need to focus more on their breathing before starting the TS portion of therapy, then one or more of the above steps would be repeated. 

Therapeutic Singing (TS)

This final and most advanced step of the three-component NMT technique combines all the above-learned respiratory exercises in a song format. The therapist selected each participant’s preferred genres, songs, or artists to optimize motivation and participation. Using an individual’s preferred music genre may benefit mood, reduce anxiety, and make the therapy process more engaging. While singing or producing vocal sounds (even off-tune), participants were encouraged to hold each musical phrase in one breath, thereby building upon components of DB and VIT learned earlier. If the person could read without struggling, they would be provided the lyrics. If persons could not read due to new or pre-existing conditions, they were then encouraged to sing as much as they could from memory. The therapist and available intern sang along with each person to make them more comfortable singing and avoid any sense of performance anxiety as if they were singing a solo. Participants would often remember the lyrics to a preferred song once they heard the song begin. 

Participant experience data collection

A music therapy intern recorded outcome data for the primary music therapist throughout the protocol. Two music interns were involved, one for each academic year in which the study took place. In the final NMT session, the interns asked each participant about their overall experience with the music therapy program using a standard questionnaire.

Individual responses were categorized into positive, negative, or undetermined separately for respiratory and breathing impact and psychological experience. In terms of respiratory and breathing benefits, participants were asked to consider changes in physical exertion or effort needed for breathing and vocal quality (meaning clarity of voice). 

In addition, NMT participants were asked to rate their perceived tolerance to the program using three categories: tolerated well without program adjustments, tolerated only with adjustments of duration and intensity, or unable to tolerate without resulting in post-exertional exhaustion. Severe fatigue the following day suggested prolonged recovery time, which in turn impacted the subsequent day’s therapy progress. If a patient had an unexpected discharge, attempts were made to obtain patient experience comments shortly after discharge during a phone call to review standard discharge follow-up. Because the goal of evaluating patient experience outcomes was to improve the center’s NMT program specifically, rather than gather feedback on all therapy programs offered, data were only obtained from NMT participants. There was no comparable information gathered from non-NMT participants.

Statistical analyses

Descriptive statistics (e.g., frequencies, means, standard deviations) were calculated for sample characteristics. Pearson correlations were used to determine the association between continuous outcomes and demographic variables. To assess mean differences between groups, Student's t-tests were conducted. To assess differences among the NMT participant group at admission and discharge, a non-parametric Student's t-test was done. Cohen’s d was calculated to assess the effect size of the intervention between groups for statistically significant continuous outcomes. We used the standard determination of the quality of the effect size: .2 was considered a small effect, .5 was considered a moderate effect, and .8 was considered a large effect. Chi-square tests were conducted for categorical outcomes. For all statistical analyses, a relationship was considered statistically significant at the < .05 level.

In situations of missing data on final phonation or patient experience, participants were dropped from the specific analysis for which findings were absent. Due to the small sample sizes in each group, no subgroup interactions were conducted.

## Results

**Figure 1:**
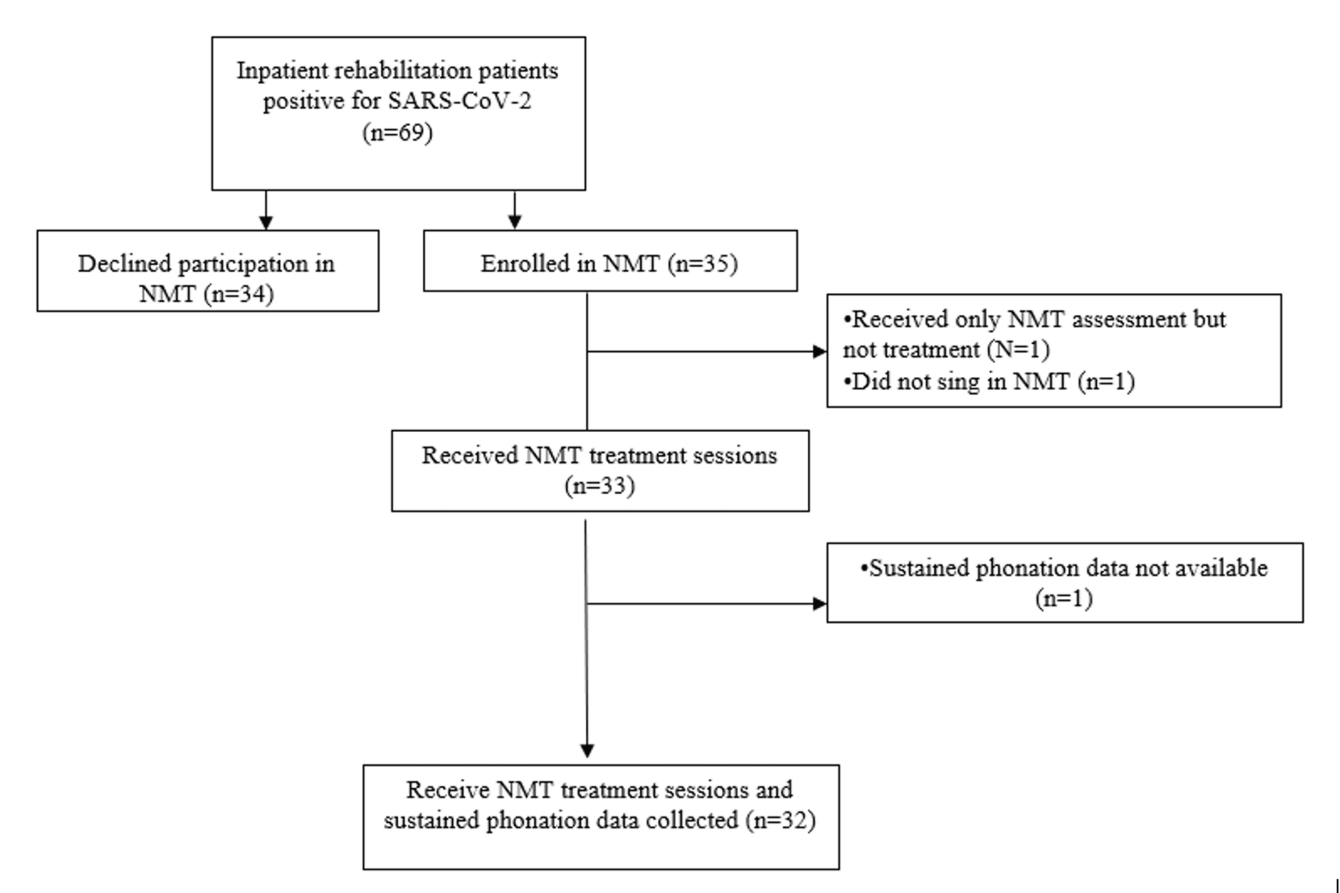
Participant inclusion flow diagram NMT: neurologic music therapy

A total of 69 participants between the ages of 29 and 88 took part in the study. Thirty-five people who met the inclusion criteria elected to receive individual NMT sessions to improve breathing function following recent acute COVID-19 or PASC. Two participants initially enrolled in NMT were eliminated. One was withdrawn before treatment began and the other declined to sing but listened to the music, thus leaving 33 individuals. One participant lacked final phonation data but had other outcomes reported. Thirty-six participants declined NMT. Figure 1 shows an inclusion flow diagram of participants who were referred for NMT and those who did not desire NMT. Reasons for non-participation included patient or family preference, the decision of the physician that other non-respiratory medical issues would preclude enrolment, and the anticipated length of stay being too short for meaningful gains. A history of migraines was noted in several charts of nonparticipants as one reason for declining NMT. More Black individuals were in the non-NMT treatment group than in the NMT group, but this difference was not significant. Several Black individuals had sung in groups or choirs before becoming ill and stated that engaging in this activity would merely reinforce another aspect of the life they had lost. They believed partaking in NMT treatment would result in greater anxiety or depression. 

Table 2 lists the participants' characteristics and pre-existing medical comorbidities. Time between the initial positive COVID test and admission to rehabilitation was more than twice as long among NMT participants than in those not receiving NMT (p<.001). Despite this difference and greater oxygen use among the NMT group both at the time of discharge and throughout their rehabilitation stay (Table 3), a significantly higher percentage of NMT participants were discharged home (97.0% vs. 77.8%, p=.018, Table 2). However, the number of days in rehabilitation was similar between groups. No follow-up was done after discharge.

**Table 2:** Participant background characteristics *Statistically significant difference NMT: neurologic music therapy; NA: Too few cases in cells to run a test of differences between groups Discrete variables listed as n (%).

Characteristics	Did Not Receive Neurologic Music Therapy Treatment (n=36)	Received Neurologic Music Therapy Treatment (n=33)	p-value
Average age (years)	66.6 (SD +/- 16.3)	61.7 (SD +/-14.6)	.189
Sex			
Female	21 (58.3%)	12 (36.4%)	.068
Male	15 (41.7%)	21 (63.6%)	
Race			
Black	14 (38.9%)	7 (21.2%)	.475
Caucasian	22 (61.1%)	26 (78.8%)	
Ethnicity			
Hispanic	1 (2.8%)	4 (12.1%)	NA
Non-Hispanic	35 (97.2%)	29 (87.9%)	
COPD	12 (33.3%)	6 (18.2%)	.152
Asthma	10 (27.8%)	5 (15.2%)	.204
Diabetes	14 (38.9%)	13 (39.4%)	.966
Obesity (BMI > 25)	13 (36.1%)	16 (48.5%)	.298
Current Tobacco Smoker	4 (11.1%)	4 (12.1%)	.903
Ventilator Use Before Admission	12 (33.3%)	16 (48.5%)	.200
Average Length of Stay for Inpatient Rehab (days)	20.0 (SD +/- 9.4)	18.9 (SD +/- 8.9)	.632
Average Number of Days Between First Positive COVID-19 Test and Rehab Admission	18.9 (SD +/- 21.3)	47.3 (SD +/- 33.0)	< .001*

**Table 3:** Differences in rehabilitation outcomes between non-NMT and NMT participants *Statistically significant difference NMT: neurologic music therapy; IP: inpatient; NA: too few cases in cells to run test of differences between the groups

Outcome	Non-NMT group (n = 36)	NMT group (n = 33)	p-value
Average number of days between first positive COVID-19 test and rehab admission	18.9 (SD +/- 21.3)	47.3 (SD +/- 33.0)	< .001*
Average length of stay for inpatient rehab (days)	20.0 (SD +/- 9.4)	18.9 (SD +/- 8.9)	.632
Discharge Setting: Home	28 (77.8%)	32 (97.0%)	.018*
Tracheostomy at Rehab Admission	3 (8.3%)	5 (15.2%)	.071
Tracheostomy at Rehab Discharge	1 (2.8%)	3 (9.1%)	NA
Any Supplemental Oxygen Use:			
At IP Rehab Admission at Rest	15 (41.7%)	18 (54.5%)	.285
At IP Rehab Admission With Activity	15 (41.7%)	23 (69.7%)	.019*
At IP Rehab Discharge at Rest	7 (19.4%)	11 (33.3%)	.189
At IP Rehab Discharge With Activity	9 (25.0%)	17 (51.5%)	.023*
Average Amount of Supplemental Oxygen Use			
At IP Rehab Admission at Rest	1.06L (+/-1.47L)	1.65L (+/-1.73L)	.128
At IP Rehab Admission With Activity	1.19L (+/-1.86L)	2.64L (+/-2.63L)	.010*
At IP Rehab Midpoint at Rest	0.61L (+/-1.20L)	1.50L (+/-1.82L)	.019*
At IP Rehab Midpoint With Activity	0.86L (+/-1.33L)	2.39L (+/-2.42L)	< .001*
At IP Rehab Discharge at Rest	0.49L (+/-1.18L)	0.91L (+/-1.47L)	.095
At IP Rehab Discharge With Activity	0.68L (+/-1.37L)	1.82L (+/-2.28L)	.007*
Tachycardia Limiting Therapy			
At IP Rehab Admission	5 (13.9%)	7 (21.2%)	.490
At IP Rehab Midpoint	3 (8.3%)	5 (15.2%)	NA
At IP Rehab Discharge	2 (5.6%)	3 (9.1%)	NA

Most participants required some supplemental oxygen support at the time of rehabilitation admission (Table 3). There was a significantly higher percentage of supplemental oxygen use during activity in the NMT group than in the non-NMT group (69.7% versus 41.7%, p=.019). Both groups saw a decrease in average supplemental oxygen requirements halfway through inpatient rehabilitation and at discharge, but significantly fewer members of the non-NMT group required oxygen at rehabilitation admission. NMT participants stayed longer in acute care and arrived at rehabilitation needing more oxygen support than non-NMT individuals. Supplemental oxygen may have been used for only a portion of the day or evening, but precise hours of daily use per patient were unavailable in the medical record. Within this limitation of available data, greater supplementation was required to maintain necessary oxygen saturations in the NMT group than in the non-NMT group. Significant differences in oxygen need between groups were seen not only at the time of admission to rehabilitation but also at mid-stay and discharge.

While the degree of relative improvement seen with oxygen use at rest between groups was not substantial, oxygen use during activity was significantly different, with the NMT group showing better gains (p =.014, Cohen’s d 2.069). At the time of discharge, NMT participants used 0.82 L less oxygen compared with the use at admission, while the non-NMT group only improved by 0.51 L of oxygen. Confidence intervals and effect sizes using Cohen’s d are given for all values that were significant in Table 4.

**Table 4:** Mean differences in oxygen use between groups for outcomes IP: inpatient

Average amount of supplemental oxygen use	Mean Difference	95% CI lower	95% CI upper	Cohen’s d
At IP rehab admission at rest	-.5960	-1.3670	.1751	1.6028
At IP rehab admission with activity	-1.442	-2.531	-.353	2.264
At IP rehab midpoint at rest	-.8889	-1.6242	-.1536	1.5286
At IP rehab midpoint with activity	-1.533	-2.409	-.657	1.821
At IP rehab discharge at rest	-.4230	-1.0599	.2140	1.3241
At IP rehab discharge with activity	-1.1376	-2.0337	-.2416	1.8628
Change in oxygen use from rehab admission to discharge at rest	.145	-.4350	.7254	1.2061
Change in oxygen use from rehab admission to discharge with activity	-1.117	-2.1126	-.1223	2.069

To better evaluate the role of NMT in breathing recovery, we isolated the NMT participant data for changes in oxygen demand throughout rehabilitation. A non-parametric Student's t-test was done to explore decreased oxygen needs from rehabilitation admission to discharge, both during times of rest and during activity. Significant differences were found in both analyses. Specifically, at rest, oxygen demand declined by .7833L (p=.003) and during activity, oxygen need decreased by .8500L (p=.003).

We had initially thought that the use of vibratory stimulation in combination with regular respiratory treatments might add to the therapeutic effect of NMT. While no differences between groups were observed in the combined effects of vibratory stimulation and NMT, some participants reported an easier ability to expectorate secretions following music training sessions (see patient experience section). Persons unable to fully expectorate any secretions would use the vibratory stimulator to help remove deeply retained mucus in smaller airway passages. Although the combination of both measures may have helped individual cases, no quantifiable outcome on the effect of either intervention could be determined. Of the 33 participants in the final NMT group, 15 reported increased mucous production following individual NMT sessions (3 with and 12 without tracheostomy tubes).

Our second main outcome measure of phonation utilized DB and VIT but not TS. There was a strong correlation between the number of treatment sessions and change in phonation, with more sessions of NMT correlating with the ability to phonate for longer periods of time (Pearson correlation 0.405, p=.021). In examining the relationship between treatment minutes and change in phonation, the correlation was even greater. More time correlated strongly with longer phonation (Pearson correlation 0.454, p=.009). Treatment minutes and average number of sessions are given in Table 5.

**Table 5:** Phonation outcomes IP: inpatient

	Mean (Standard Deviation)
Minutes of Neurologic Music Therapy Treatment	174 (SD +/- 114)
Number of Neurologic Music Therapy Sessions	4.2 (SD +/- 2.33)
Sustained End Phonation at IP Rehab Admission in seconds	9.7 (SD +/- 4.8)
Sustained End Phonation at IP Rehab Discharge in seconds	14.7 (SD +/- 6.9)

Participant experience outcomes

Participants were asked to rate their overall experience with the NMT program for breathing and respiratory function and separately, for any psychological benefits. Of 33 NMT participants, 30 completed final patient experience assessments. The three missing outcomes were due to either early discharge or schedule changes limiting the final session when this data would have been obtained. For breathing and respiratory function, individuals were asked to consider how NMT altered their effort in breathing and speaking, their “vocal stamina,” and their ability to speak words in a sentence with fewer pauses to breathe. They were also asked to consider if NMT improved their clarity of voice (less hoarseness, less muffled sound). In the respiratory outcome measure, 76% rated their experience as positive, but 9% (3 people) rated the experience as negative.

The music therapist conducting this study reported that three people who participated in NMT continued to need a tracheostomy tube while in rehabilitation. These individuals were observed to expectorate more secretions just after the DB and VIT exercises. Two of three participants felt this vigorous clearance of mucus was a negative effect of the therapy when later asked to rate their participant experience and the overall value of NMT. However, the third participant with a tracheostomy felt that the NMT aided in vocal strength, and this individual perceived mucus clearance as a benefit. One non-trach NMT participant also rated the experience as negative for unclear reasons. Of those without tracheostomy tubes, a chart review indicated 12 of 30 also noted increased coughing with mucus clearance immediately following an NMT session. These 12 participants believed that the NMT had a positive effect on vocal quality due to the elimination of thick secretions that had muffled their voice and prompted the need to clear their throat by coughing intermittently.

When designing the NMT protocol, the music therapy staff initially expressed concerns about fatigue and endurance in persons with relatively recent COVID-19 and those with PASC. Recovery of pulmonary function can take months before patients feel the same energy they possessed prior to their prolonged illness. In 18.2% of cases, adjustments to the NMT protocol were needed due to fatigue or pacing, given three hours of standard rehabilitation therapies. With modifications, this group was able to proceed with NMT, resulting in an overall positive experience for breathing function. However, in 9.1%, modifications were insufficient. This group noted post-exertional fatigue the day following music training sessions, adversely impacting their other therapies. Consequently, this group reported a negative experience with NMT for breathing function.

Approximately 82% of patients made positive statements regarding their psychological experiences in music therapy. Nine percent said the experience was negative and the remainder were rated as undetermined because no response could be obtained. These are summarized in Table 6.

**Table 6:** Patient experience with NMT for respiratory function and psychological benefit Breathing/respiratory experience: Positive 76%, Negative 9%, Undetermined 15% Psychological experience: Positive 82%, Negative 9%, Undetermined 9% NMT: neurologic music therapy

Question asked to NMT participant	Yes (n/%)	No (n/%)	Undetermined (n/%)
Did you feel the NMT program improved breathing and respiratory function? (improved ease of breathing or speaking, more words spoken per breath, improved vocal clarity)	25 (76%)	3 (9%)	5 (15%)
Would you rate the NMT program as a positive psychological experience?	27 (82%)	3 (9%)	3 (9%)

To better illustrate how participants felt about NMT, additional subjective comments to the music therapy team were collected. Although not required in the protocol, some participants wanted to add additional feedback to the team. Their thoughts included:

 “I have been in this hospital SO LONG, now over 100 days, I just want to go home. I am less panicked about getting out of breath after doing this music therapy, I don’t feel like I’m gasping for air anymore, but no way am I 100%.”

“The reason I signed up for this [music therapy] was to get out of my room, but I seriously doubted it would help change anything. But actually, this therapy is helping get the thick stuff out of my lungs and I am now able to talk without clearing my throat every two seconds.”

“This is much harder than PT. But I can see why you ordered this. It definitely helps get phlegm up. I feel my breathing is not as much of a struggle, but I am getting exhausted. These lessons are too long. Can you cut them in half?”

## Discussion

Music therapy is a non-invasive, comparatively safe, and inexpensive adjunct to standard therapies offered during inpatient rehabilitation. While many people enjoy music, not all enjoy singing [3]. In this protocol, NMT served as a tool to improve breathing stamina and vocal ability with the goal of increasing speaking time with reduced effort. Singing per se was neither an aim nor an expectation. When used to enhance vocal function, sequential strengthening exercises for vocal output may help reinforce the synchronized interaction of breathing through the use of key respiratory muscles and accessory muscles of respiration, phonation, and resonance [7,19]. Continued practice of a daily exercise regimen that coordinates key features of vocal production appears to improve efficiency and may lessen the fatigue of speaking. Our findings demonstrate improved phonation, measured in seconds, with continued minutes of therapy and additional sessions of NMT. The efficiency of voice production, the mechanical process of actual singing, and the ability to sustain tones for increasingly longer periods of time are all based on training. In accomplished singers, vocal ability is also based on the experience of the singer, but the techniques used by professionals for optimizing vocal training can help those with weak voices and breathing challenges to become stronger [20].

Oxygen use

Findings demonstrated an average reduction in oxygen needs from admission to discharge for the NMT participants at rest and with activity. NMT could represent one contribution to the reduction in oxygen, although both the time with increased medical stability and the contribution of other therapies likely contributed synergistically. Moreover, while the change in oxygen use in Liters was statistically significant, clinical significance is unknown.

Music therapy and mucus clearance

Patients who participated in NMT had individual instruction for an average of 45 minutes per session, with direct feedback. Active learning with frequent cues and repetition was an integral part of the therapy. Given many patients in the early recovery period of COVID-19 and in phases of PASC have confusion and “brain fog,” the use of breathing techniques to help clear secretions can be confusing and frustrating. We found that some patients needed individual supervision for each use of the vibratory stimulation hand-held device. Initially, we tried some other breathing therapy techniques for mucus clearance, but the complexity of maneuvers like the Huff cough was too challenging for many [8].

The participants reported having an easier time coughing up secretions that were deep in their lungs. Following the expectoration of the secretions, participants felt their voice production was less muffled and nasal-sounding. Reasons for the easier passage of secretions were unclear but may be found in the property of muscle viscosity, defined as the rate at which muscles contract. Vocal warm-up exercises, after which the VIT portion of our protocol is patterned, have a warming effect on the muscles of the respiratory system. As the temperature of the muscles increases through NMT, mucus viscosity decreases and elasticity improves, making expectoration of deep-seated phlegm easier to achieve [21]. The increased temperature also facilitates greater release of oxygen from hemoglobin, while simultaneously decreasing oxygen demand [20]. These measures may explain how NMT can improve both endurance and phonation (breath support) in our study group. In addition, the improved mucus clearance and strength may have indirectly contributed to the reduction in oxygen demand among the NMT group.

Emotional benefits of music therapy

Numerous investigations have explored the psychological and hormonal benefits of music therapy [2,22,23]. Sun et al. conducted a comparison of adults in singing versus non-singing groups [23]. Findings showed significant increases in resilience, with notably fewer adults classified as “depressed” in the singing group. The singing group also reported a better quality of life, a sense of kinship with others, and greater degrees of social support. The health benefits of singing can be linked to increases in neuropeptides and hormones as well as decreases in cortisol and stress in situations like singing for pleasure or practice, but not during a professional performance [24]. Fancourt et al. conducted another study that linked decreases in cortisol to reduced glucocorticoid suppression of the immune response [25].

Oxytocin has been shown to improve several social behaviors and psychological measures [2]. Kreutz et al found higher levels of oxytocin in choir singers as opposed to a non-singing group of comparably aged persons [22]. Several investigators have suggested that oxytocin is linked to improved levels of trust, interpersonal bonding, and reduced negative thought processes [26,27].

Secretory immunoglobulin A (S-IgA) is an antibody in the upper airway thought to protect from bacterial and viral infection. In the setting of COVID recovery, activities such as singing that increase S-IgA are welcome additions to the treatment plan. Higher S-IgA levels were found in singers of music relative to listeners of music by Kreutz et al. [22]. Similar findings by Kuhn et al. support the concept of the robust production of S-IgA occurring with active singing and instrument playing, but notably lesser S-IgA amounts were produced after passively listening to music [28]. In our study, over 80% of participants in the NMT protocol considered the program to be a positive experience. While oxytocin and other hormonal levels were not measured, enhancement of these substances could have contributed to the positive outcome in some fashion.

Two recent investigations have illustrated the benefit of music therapy on mood in those with PASC who are experiencing new-onset anxiety or depression. An RCT by Giordano et al. found that patients given receptive music therapy had significantly lower scores for anxiety and heart rate and better levels of oxygen saturation [29]. Their protocol involved only passive listening, rather than active use of vocal muscles, but the concept of music to reduce emotional stress is meaningful for the future expansion of our pilot study. In a second RCT by Hasina et al., after six short sessions of listening to music of their preferred genre, participants showed a significant decrease in self-reported scores of anxiety and an increase in self-reported quality of life [30]. Continuing to provide TS in our protocol, even if not functional for many patients, may assist with the psychological benefits of music therapy, independent of their effect on breathing function. 

Study limitations

The retrospective chart review methodology has many limitations that impact the certainty of several associations found in this investigation. The small sample size meant that a subgroup analysis was not feasible. At the outset, our groups were not equivalent. They differed in their requirements of oxygen on admission to rehabilitation and in their days spent in acute care. Given the compromised medical stability of the participants, their physical and mental fatigue, and the anticipated difficulties with their concentration and attention while reading a detailed consent form, a retrospective chart review was the most feasible means of data acquisition. In addition, controlling all the variables needed for better outcome comparisons was not possible during the height of the pandemic. Since NMT was part of the participants’ treatment program, this study was not a randomized clinical trial with a control group.

Our groups were based on voluntary participation in NMT and nonparticipation by choice. Not every NMT individual received the same number of minutes in music therapy. Music therapy was provided by three different instructors with varying levels of experience, but all sessions were conducted under the supervision of the same board-certified neurologic music therapist. Treatment time was based on each individual’s length of stay and availability to be in music therapy. At times, participants had medical appointments or were too fatigued to attend NMT after their other daily therapies. The NMT group had a longer acute hospital length of stay and was more medically compromised. Our inclusion dates spanned 24 months; thus, participants enrolled likely had different strains of the virus. This variability could have affected respiratory symptoms and influenced endurance and participation. There may also have been an inherent bias in the self-selected division of groups that may reflect the motivation of participants. Due to such a prolonged hospitalization, they may have been more motivated to go home and enroll in any therapeutic activity that might facilitate an earlier discharge.

This study measured expiratory strength in the form of phonation. No measurements of inspiratory strength or vital capacity were taken since NMT providers did not have the technology or tools for these measurements during normal treatment sessions, nor were these tools readily available on the rehabilitation floor during the pandemic. Equipment use was limited by concerns of cross-contamination and supply chain barriers in obtaining individual peak flow meters for each post-COVID patient. The data collection for oxygen use was limited by the staff’s ability to accurately record different oxygen uses throughout the day. Oxygen use was instead recorded at the start of each 12 or 8-hour nursing shift. Similarly, we were unable to control how faithfully participants used their vibratory stimulation hand-held devices. Making the device available at the bedside and reminders to nursing staff to monitor patients while performing these exercises were insufficient to ensure compliance with device use.

One of our outcome measures was the discharge setting. Reasons for choosing home versus skilled nursing or other settings are multifactorial and influenced by caregiver or home care availability, community resources for patient support, and distance from outpatient therapy centers. Historically, participant experience data are subjective in nature and at risk of bias.

As the years since the world first experienced COVID-19 have progressed, the nature of the virus and the features of acute and chronic illness have evolved. The NMT protocol, devised initially in 2020, occurred at a time when relatively little was known about the long-term effects of COVID-19. Given the knowledge gained over the last several years, broad application of our results to other groups would not be appropriate. Concepts involving strengthening muscles of voice production and adjustments of any music therapy protocol to the individual needs of each patient would be highly relevant to any future study. However, given the limitations of our investigation, this pilot study serves primarily as a starting point for larger, prospective trials.

For future protocols, oral motor and respiratory exercises (OMREX) could be considered for inclusion in NMT. OMREX is a technique for addressing the improvements of articulatory control, respiratory strength, and function of the speech apparatus [4]. Singing materials and exercises are applied to enhance articulatory control and the respiratory strength and function of the speech apparatus [7]. OMREX exercises can be achieved with singing as well as playing wind instruments. Singing can have the added benefit of assisting the general respiratory function, which can improve the patient’s ability to complete activities of daily living and enhance their quality of life. Rhythm can help set the rate of breathing frequency and depth, properties that augment both breath control and strength [3]. Techniques involved in OMREX can be used for breathing exercises aimed to enhance vocal strength and laryngeal function that further enrich respiratory capacity and oral motor function [4].

## Conclusions

This pilot investigation demonstrated three central outcomes. First, an NMT protocol may help reduce reliance on oxygen both at rest and during exertion when used as an adjunct to standard respiratory interventions. Second, a correlation exists between phonation or breath support during vocal exhalation and time in NMT sessions. Third, participants had a positive impression of NMT in assisting with respiratory and breathing function and in improving psychological outlook.

The NMT procedure in this report significantly improved sustained phonation and breath control in people with early recovery from SARS-CoV-2 infection, as well as PASC. These findings may have direct implications for patients’ ability to speak longer phrases or complete sentences without stopping mid-thought to rest or take more breaths. Ease of mucus clearance reported by participants may be an added benefit of music therapy for those with tracheostomy tubes or retained mucus from prolonged illness. Despite a significantly greater need for supplemental oxygen at discharge, those who received NMT went home at a notably higher rate. Moreover, the great majority of NMT participants reported that the experience was both feasible and psychologically beneficial. Given its many positive features, NMT should be considered a low-cost, low-risk adjunct to respiratory therapy in persons recovering from COVID-19.
